# Canada's Neglected Tropical Disease Research Network: Who's in the Core—Who's on the Periphery?

**DOI:** 10.1371/journal.pntd.0002568

**Published:** 2013-12-05

**Authors:** Kaye Phillips, Jillian Clare Kohler, Peter Pennefather, Halla Thorsteinsdottir, Joseph Wong

**Affiliations:** 1 Leslie Dan Faculty of Pharmacy, University of Toronto, Toronto, Ontario, Canada; 2 Dalla Lana School of Public Health, University of Toronto, Toronto, Ontario, Canada; 3 Department of Political Science, University of Toronto, Toronto, Ontario, Canada; Oswaldo Cruz Foundation (FIOCRUZ), Brazil

## Abstract

**Background:**

This study designed and applied accessible yet systematic methods to generate baseline information about the patterns and structure of Canada's neglected tropical disease (NTD) research network; a network that, until recently, was formed and functioned on the periphery of strategic Canadian research funding.

**Methodology:**

Multiple methods were used to conduct this study, including: (1) a systematic bibliometric procedure to capture archival NTD publications and co-authorship data; (2) a country-level “core-periphery” network analysis to measure and map the structure of Canada's NTD co-authorship network including its size, density, cliques, and centralization; and (3) a statistical analysis to test the correlation between the position of countries in Canada's NTD network (“k-core measure”) and the quantity and quality of research produced.

**Principal Findings:**

Over the past sixty years (1950–2010), Canadian researchers have contributed to 1,079 NTD publications, specializing in Leishmania, African sleeping sickness, and leprosy. Of this work, 70% of all first authors and co-authors (n = 4,145) have been Canadian. Since the 1990s, however, a network of international co-authorship activity has been emerging, with representation of researchers from 62 different countries; largely researchers from OECD countries (e.g. United States and United Kingdom) and some non-OECD countries (e.g. Brazil and Iran). Canada has a core-periphery NTD international research structure, with a densely connected group of OECD countries and some African nations, such as Uganda and Kenya. Sitting predominantly on the periphery of this research network is a cluster of 16 non-OECD nations that fall within the lowest GDP percentile of the network.

**Conclusion/Significance:**

The publication specialties, composition, and position of NTD researchers within Canada's NTD country network provide evidence that while Canadian researchers currently remain the overall gatekeepers of the NTD research they generate; there is opportunity to leverage existing research collaborations and help advance regions and NTD areas that are currently under-developed.

## Author Summary

This study applies co-authorship network analysis to generate baseline information about the patterns and structure of Canada's neglected tropical disease (NTD) publication activity and research network. Researchers, public and private funders, not-for-profit organizations, and policy makers may use the methodology or study findings for targeting, monitoring, and assessing Canada's contribution to a research field that is ready for attention and advancements. Future studies could use the findings to comparatively analyze the emergence of specific NTD research amongst institutional networks or further examine attributes and mechanisms that support and impede Canadian involvement in NTD research production and collaborative North–South research partnerships.

## Introduction

The cadre of research and development focused on neglected tropical diseases is driven by a common mission: generating discoveries, treatments, and interventions that will help reduce the global burden of a significant group of communicable diseases that thrive in impoverished settings and affect over 1 billion of the world's 2.8 billion poorest people. Collaborative neglected tropical disease research and development (where researchers work together from across disciplines, institutions, sectors, and countries) is as a strategy increasingly used to mobilize technical resources and diffuse the liability and financial risks amongst researchers and institutions involved in developing and bringing innovative solutions to market. It's also used to ensure research agendas are driven by the needs and priorities of low-and-middle income countries.

While collaborative research networks have many attributes and characteristics, they can loosely be defined as formal or informal networks of individuals and organizations that are working together and share similar mandates, goals or activities [1], [2]. Formal research networks are typically established to meet specific organizational or policy goals (e.g., building research capacity; encouraging connections between researchers and users; building multi-disciplinary research agendas) within specific fields. Globally, a surge of formal collaborative NTD research and development networks in the form of public private partnerships (PPPs) and product development partnerships (PDPs) have been emerging over the past ten years. The Drugs for Neglected Diseases Initiative (DNDi), the Institute for One World Health (iOWH); the Pediatric Dengue Vaccine Initiative (PDVI); the Human Hookworm Initiative (HHI); Rotavirus Accelerated Development and Introduction Plan; and Pneumococcal Vaccine Accelerated Development and Introduction Plan are initiatives that each involve formal commitments from a collective of organizations, from across sectors, working to generate and deliver NTD research and development solutions. Although promising for generating innovation and economic growth, collaborative research initiatives such as PPPs and PDP have also been criticized as serving private-industry interests and intended to boost industry public relations and access to human resources and financial gains from vulnerable markets [3], [4].

On the contrary, informal research networks are often characterized as dynamic and responsive collectives of researchers that tend to evolve on the periphery of formality and structure; often a result of researchers who partner with other researchers based on common and complimentary interests, expertise, and access to resources in a specific field [1], [5]. Literature related to informal NTD research networks has largely been published in the form of bibliometric co-authorship mapping studies and is increasingly emerging to inform critical dialogue and commentary about the role of publicly funded researchers and institutions in ensuring under-resourced regions have more equitable access to NTD innovations and delivery solutions [6], [7]. Informal research networks are important, as their adaptability and ‘grass-root attributes’ point to the nexus of innovation and the opportunity for research funding agencies to identify and target mechanisms (financial and non-financial) to best harness their performance and ensure the effective use of resources and research investments.

Underlying the endorsement of collaborative research initiatives is the assumption that they will involve co-operative practices that result in strengthened productivity, quality, and relevance for all parties concerned. Although research that is conducted collaboratively has been identified as a means for multiple actors to access and produce new knowledge, gain peer recognition, and acquire professional opportunities and intrinsic awards [8], [9]; the extent to which neglected tropical disease research is driven by and takes advantage of the brain-power of low-and-middle income country experts and infrastructure is up for debate and a criticism of international and philanthropic collaborative research initiatives [10], [11].

Within Canada, while the area of NTDs has not been a strategic priority area for its research granting councils, in recent years there is indication of an emerging formal research network (through initiatives such as the Canadian Institute for Health Research (CIHR) Chair in Neglected Diseases and Grand Challenges Canada Rising Star winners) and an active informal Canadian NTD research community [12]. However, there exists a challenge and deficiency in generating evidence to understand the structure and demonstrate the equity, effectiveness, outcomes, and evolution of NTD research and development and research networks, domestically and on a global scale [13]–[15].

In an effort to generate evidence about Canada's NTD publication activity and the structure of its research network, this study conducted a country-level co-authorship network analysis. Accessible yet systematic methods were designed and applied to generate a baseline for tracking and measuring patterns and trends in the production, specializations, composition, and position of researcher in Canada's NTD research network. Archived co-authorship publication data were used, as a proxy for collaboration, to generate country-level network analysis measures (including components and size, density, cliques, and centralization). The data were further analyzed by examining the core-periphery structure of Canada's NTD co-authorship network and testing the correlation between the participation of countries in collaborative publishing (groups) against the quantity and quality of publications. Understanding the structure of a network makes the grouping and position of its actor's visible (individuals, institutions, or countries); and helps expose potential gaps within the network. Evidence increasingly suggests that the position of actors within a network influences and shapes the production, practice, and diffusion of research [16].

## Methods

### NTD Keyword Classification

In this study neglected tropical diseases are delineated as including diseases that cause significant morbidity and mortality in poor and rural populations but are the most severely neglected in terms of basic research, development, and deployment of safe and effective interventions [17]. The NTD list generated by Hotez et al (2006) was adopted for this study to focus the inclusion criteria and its NTD keyword classification. Hotez et al's list is widely accepted as including the diseases with the most prevalent impact and includes the thirteen NTDs commonly known as roundworm, whipworm, hookworm, snail fever, elephantiasis, blinding trachoma, river blindness, Leishmania, Chagas disease, leprosy, African sleeping sickness, Guinea worm, and Buruli ulcer. Although there are slight nuances in how organizations and researchers define and categorize NTDs, they are commonly distinguished from the big three infectious diseases (HIV/AIDS, tuberculosis, and malaria), which generally receive significantly more research and development funding [12], [17].

A three-category classification was then developed for each NTD to ensure that a comprehensive search using appropriate terminology could be completed. Each NTD was classified by the common name(s); the scientific disease name(s); and the name of the disease agent. The World Health Organization (WHO) International Classification of Diseases [18] and the NIH-National Library of Medicine [19] informed the nomenclature development of the three-category classification and Scopus search string that was developed for the twelve NTDs (File S1). For the scope of this study, the scientific classification of the disease agent was limited to the genus and species. This classification was appropriate, given that the disease pathogen is invariant and must be a part of a research-driven solution.

### Database Mining

Archived co-authorship publication data are increasingly used to understand scientific production and research collaborations [20]–[23]. NTD publications that included Canadian first authors or co-authors (defined as at least one author whose institution is affiliated with a Canadian address) over a sixty year period (1950–2010) were gathered as raw data using the Scopus database. For the scope of this study co-authorship data was collected on authors one to nine. Scopus is a major multidisciplinary database for the social sciences, life sciences, health sciences, physical sciences, and arts and humanities and includes nearly 18,000 titles from more than 5,000 international publishers. In the field of tropical medicine, evidence suggests that Scopus includes 55% more papers on tropical medicine than the Thomson Reuters ISI Web of Knowledge database and has the largest selection of journals from more countries and with a greater variety of fields [24], [25]. It also allows a search of the addresses of all contributing authors.

In order to run the search, the keyword taxonomy and search strategy for each of the thirteen NTDs was employed using the “TITLE-ABS-KEY” command, which searches all publication titles, abstracts, and keywords. To yield research authored or co-authored by researchers with a Canadian institutional affiliation, the AFFILCOUNTRY command was also used. Due to resource limitations, the scope of this study was limited to English-language scholarly work including peer-reviewed publications, publications in press, and peer-reviewed conference papers. Implications of these inclusion criterion are further described in the discussion section.

### Exporting Procedure and Screening Criteria

An abstract inclusion-screening criteria was developed and tested by reviewers (n = 2) on a subset of publications. Duplicates were removed, the dataset of publications was screened, and publications were excluded from the study if they were: 1) not authored or co-authored by a researcher affiliated with a Canadian university or institution, 2) not focused on one of the thirteen identified neglected tropical diseases, or 3) non-scholarly publications (Table 1).

**Table 1:** Publication screening results.

# of publications from initial search	2,063
# of duplicates removed:	205
# of non-NTDs removed:	391
# of non-scholarly publications removed:	311
**Total # of Publications:**	**1,079**

### Standardizing and Coding Co-Author Attributes

The bibliometric data were standardized and coded by hand in order to correct misspellings and ensure consistency between author institutions and countries. A country codex was developed to categorize and analyze co-authors by country, world region continent, OECD status, and Gross Domestic Product (using the 2009 International Monetary Fund, World Economic Outlook Database).

### Network Visualization and Analysis

The co-authorship data generated through the Scopus bibliometric search strategy were formatted and run using UCINET and NetDraw software. The software UCINET is a network analysis tool that reads the matrices and performs statistical analysis of the whole network or measures of relationships within a network. The network metrics were calculated using the UCINET formulas, saved as both Excel and ##h files and exported to NetDraw for visual analysis. NetDraw is software that is compatible with UCINET and used to assemble, visualize, and analyze various network parameters [26].

The archival NTD publication data were used to create a binary relational matrix (also known as 1-mode data) that shows the presence of relationships between the country of each author within Canada's NTD research network. Networks can be asymmetric (whereby links are directed one way) or symmetric (whereby links are undirected). Canada's NTD research network represented a symmetric (undirected) network because each link between the contributing author country was reciprocated.

In order to analyze the group relations and structural characteristics of Canada's NTD research country network, a set of network-level measures, commonly used to support macro-level analysis, were calculated and applied (size, density, cliques, centrality degree, centralization, and k-core) (Table 2).

**Table 2:** Canada's NTD network-level measures and results.

Measure	Description	Results
Size	The total number of nodes (actors) that define the unit of network analysis.	62
Clique	The set of nodes (actors) within a network all directly connected to one another. Represents a dense pocket of interconnectivity.	> = 3 members: 46> = 2 members: 42
Density	The number of connections (links) within the network as a fraction of the total number of possible connections (links).	Mean: 0.422 SD: 3.88
	D = *l*	
	n(n-1)	
	*l* represents the number of links in the network, and *n* is the network size.	
	For undirected (symmetric) networks, the numerator is multiplied by 2 –, which is the case for this study.	
Centrality Degree (CD)	The number of links a country sends and receives.	Mean: 25.74 SD: 73.97 Range: 1, 541
	The centralization of a network, the degree to which the network's ties are focused on one node (actor) or a set of nodes (actors), is indicated by the standard deviation of the centrality scores for the network.	
	A small standard deviation indicates little variation in the centrality scores and a decentralized structure.	
	A large standard deviation indicates a lot of variation in the centrality scores and a centralized structure (Valente, p. 140).	

## Results

### Canada's NTD Publication Activity and Specializations

**Figure 1:**
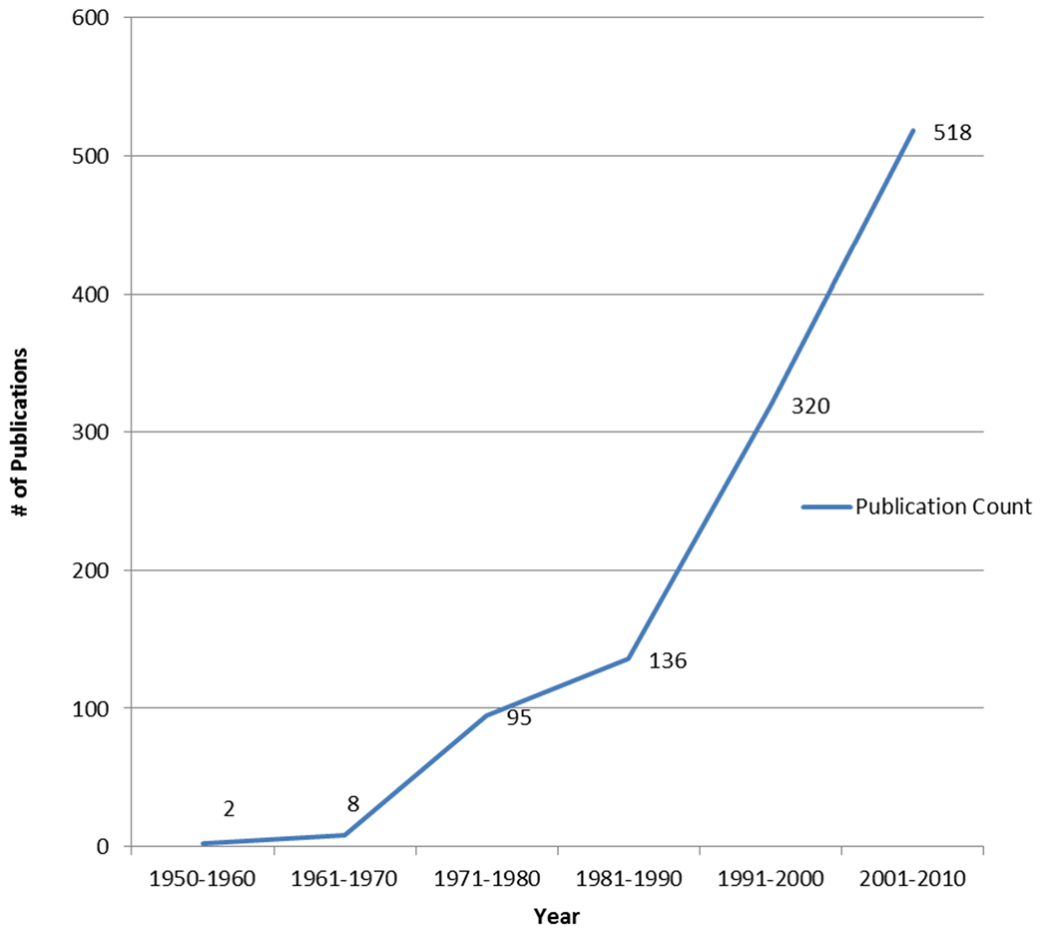
Canada's NTD research. Trends in publication growth 1950–2010 (n = 1,079).

Between 1950 and 2010, Canadian researchers contributed to the production of 1079 NTD publications as per the criterion established for this study. During this time 105 publications were produced from 1950–1980 and 974 were published from 1980–2010 (Figure 1). A majority of Canada's NTD publications were authored solely by Canadians (n = 700).

**Figure 2:**
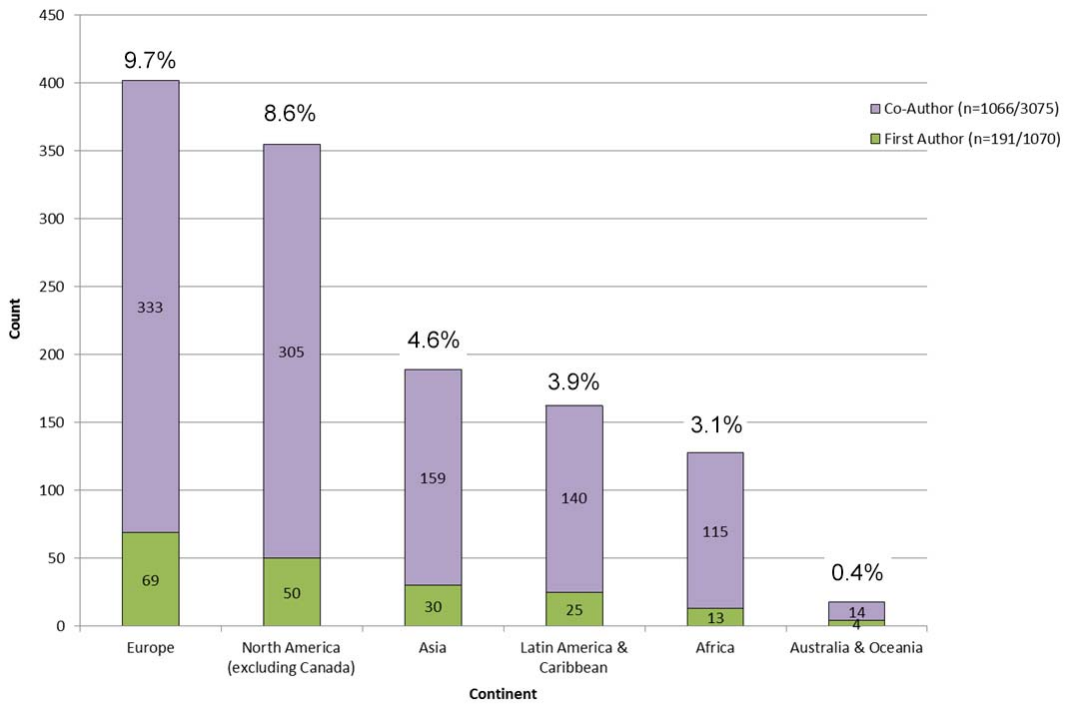
Canada's NTD research. International share of co-authors and first authors by continent 1950–2010 (n = 1,257).

Patterns in Canada's NTD country co-authorship activity are summarized in Table 3.Canada's 1,079 NTD publications included a total of 4,145 first and co-authors, of which 2,888 were affiliated with a Canadian institution (70%). Researchers from Canadian institutions represented 82% (879/1,079) of all NTD publication first author affiliation and 65% (2,009/3,075) of co-authors. The 200 papers that Canadians did not first author were largely led by authors from North America and European OECD nations, including the United States, United Kingdom, France, and Germany (Figure 2). Authors from these countries also accounted for a majority of remaining co-authoring activity (17%). The non-OECD countries with the most authorship activity (first author and co-author) included Brazil (1.5%), Iran (1.5%), Peru (0.7%), Uganda (0.6%), and Vietnam (0.6%).

**Table 3:** Canada's NTD publications: Top 12 countries by share of first authors and co-authors (1950–2010).

Country	First Author Count	Co-Author Count	Total Author Share	OECD Status (Y/N)
Canada	879	2,009	69.6%	Y
United States	50	305	8.5%	Y
United Kingdom	21	93	2.7%	Y
France	16	83	2.3%	Y
Brazil	12	54	1.5%	N
Iran	13	53	1.5%	N
Germany	11	43	1.3%	Y
Peru	4	26	0.7%	N
Switzerland	5	23	0.6%	Y
Uganda	0	28	0.6%	N
Vietnam	1	26	0.6%	N
Japan	5	21	0.6%	Y
Sweden	6	18	0.5%	Y
Remaining countries (n = 54)	47	293	8.2%	-
Total	1,070*	3,075	1	

* 9 publications did not include the country of the first author.

The vast difference in Canada's publication activity between each NTD is depicted in Table 4. Based on publication count, researchers from Canadian institutions predominantly specialize in Leishmania disease research (n = 423), which represents 40% of all of Canada's NTD publishing activity. The other NTDs most attended to by researchers from Canadian institutions include African sleeping sickness (n = 186), leprosy (n = 148), Chagas disease (n = 68), and roundworm (n = 51).

**Table 4:** Canada's NTD publication activity and co-authorship rate (1950–2010).

NTD Disease Type	# of Publication	# of publications with one or more other countries*	Country co-authorship rate
Leishmania	423	156	36.8%
African Sleeping Sickness	186	57	30.6%
Leprosy	148	31	20.9%
Chagas Disease	68	39	57.3%
Roundworm	51	12	23.5%
Blinding Trachoma	44	20	45.4%
River Blindness	44	21	47.7%
Elephantiasis	33	16	48.4%
Neglected Tropical Diseases	24	9	37.5%
Hookworm	18	6	33.3%
Whipworm	11	5	45.4%
Buruli Ulcer	10	3	30.0%
Snail Fever	10	1	10.0%
Guinea Worm	9	3	33.3%
Grand Total	1079	379	35.1%

* Includes all authors (first and co-authors). Only calculates one country instance per publication.

Of Canada's NTD publications, the diseases that appear to have the highest country collaboration rate include Chagas disease (57.3%), elephantiasis (48.4%), river blindness (47.4%), blinding trachoma (45.4%), and whipworm (45.4%) (Table 4). Although it is the disease with the highest number of Canadian NTD publications, Leishmania ranked fifth in country collaboration (36.8%). Country collaboration rate represents the percentage of Canada's NTD publications with authors from one or more other country. It includes all first and co-authors. To avoid double count, it accounts for only one instance of each country affiliation per publication. For example, if two researchers from London School of Hygiene and Tropical Medicine are co-authors with three University of Toronto researchers on a publication it would count as one instance of country collaboration.

### Measures of Canada's NTD Research Country-Network

In this study, the bibliometric data were run through UCINET to generate baseline structural measures of Canada's NTD co-authorship research network over a sixty year period, 1950–2010. This time period was selected in order to generate a historic view of the structure of Canada's entire NTD research network and to provide a starting point for identifying opportunities for subsequent analysis of sub-networks based on thematic patterns that emerge. The results identified a total of 62 countries within Canada's NTD network, including 46 ‘cliques’ that have at least three country members; and 42 cliques with at least two country members. Canada's entire NTD co-authorship network appears to have a moderately high density (42%) and centralized structure. Centralization is indicated by the high standard deviation (SD = 73.97) of the centrality degree scores reported for each country (range = 1, 541).

**Figure 3:**
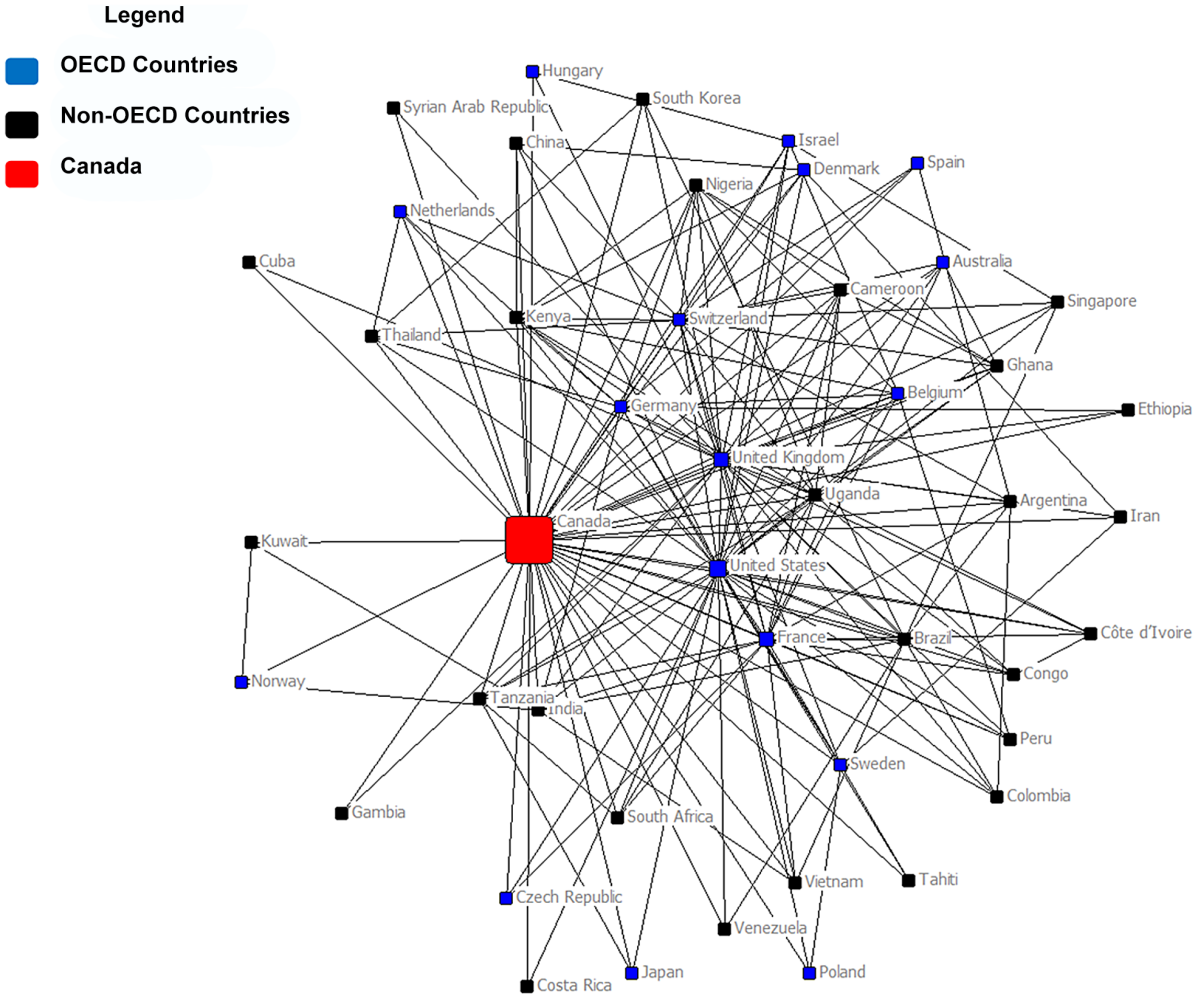
Canada's NTD co-authorship network (>2 country connections) (n = 42). Within this network map the circles (“nodes”) represent each country within Canada's NTD research co-authorship network, and the colors of the nodes represent OECD or non-OECD nations. OECD country nodes are blue and non-OECD nation country nodes are black. Canada is distinguished as a red node. The lines (“ties”) connecting one node to the next represent lines of relation between countries. The ties in this map are not valued or weighted and represent a reciprocal or undirected relation; one country either has a relation/tie with another country or not. The sizes of the nodes are scaled to represent the GDP of each country.

Figure 3 represents a multi-dimensional visual scaling of Canada's NTD research network and includes countries that have at least two country connections (n = 42). A total of sixteen countries only have a co-authorship connection with Canada and are not included in the map. These countries, which sit on the periphery of the network, are further discussed below.

### Canada's NTD Research Network: K-Core Analysis

Analyzing the size and core-peripheriness of this network (measured through k-core measures) provides insight into patterns of affiliation between collaborating countries and helps illustrate who is connected to whom within the network. Core-peripheriness is the degree to which there is a group of nodes (actors) who are densely connected (the core) and a separate group of nodes who are loosely connected to the core and each other. A k-core represents the maximal group of actors, all of whom are connected to number (k) of other members of the group [16]. A 3k-core, for instance, is the set of countries or institutions linked to at least 3 other countries or institutions. As *k* increases, the remaining actors within the network map appear increasingly dense [16].

Applying the UCINET k-core procedure on Canada's NTD co-authorship country network locates a clustering of collaborating countries in the middle right of the network map (Figure 3). The k-core measures indicate that Canada has a 7k-core NTD network where twelve countries (Canada, United States, United Kingdom, France, Germany, Switzerland, Uganda, Belgium, Kenya, Nigeria, Ghana, and Cameroon) all remain connected to each other as *k* increases from one to seven. K-core measures can identify the type of structure that characterizes a network (e.g., centralized or core-periphery).

In this country-level analysis, because the data collection was designed to always include ‘Canada’, the network would appear centralized. However, in SNA plotting, the k-core measures on a bar graph can help identify and validate the centralization of the network; by establishing whether the network has a ‘core-periphery’ structure. A core-periphery network structure indicates the variation in the number of actors in the network who are most connected to each other and those who are not. In a k-core bar graph, each bar represents the number of actors (nodes) that are dropped as each unit increases in *k*. By way of example, if Panama only has one country connection (with Canada), it would be represented in the first bar and dropped in the next. A k-core bar graph where all the bars are at the same height means that the same numbers of actors are removed at each increase in *k*. This indicates no core-periphery and little structure in the network. A k-core bar graph that steeply increases or decreases indicates structural variation (whereby the number of actors removed from the core changes abruptly). In general, the greater the percentage decrease in actors left in the core, the less a core-periphery [27].

**Figure 4:**
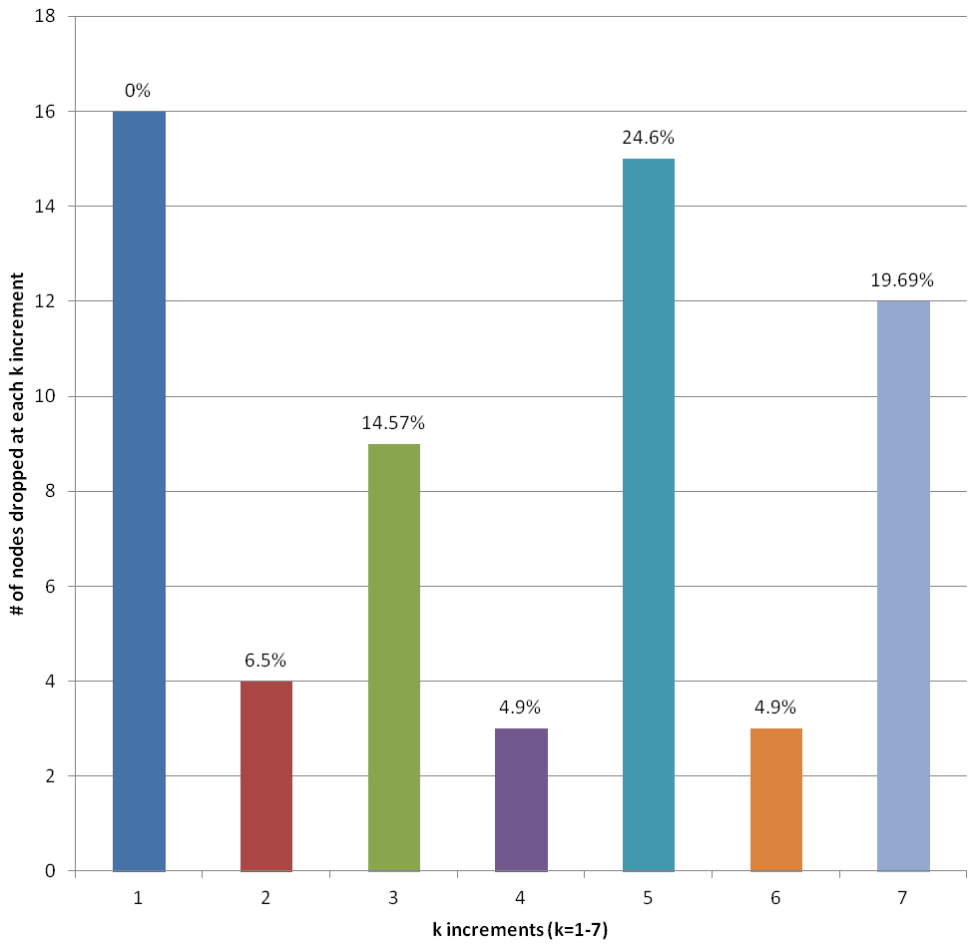
Canada's NTD research network k-core bar graph (n = 62). The country network map and the k-core bar graph show that within Canada's NTD research co-authorship network, there are dense connections among the subset of the twelve countries in the 7k-core. Therefore, there is an indication that Canada's NTD research country-level co-authorship network is a core-periphery structure. This means that there is a distinct core group of twelve countries that are all connected to at least seven other countries within the core. In contrast, there exist a number of countries on the ‘periphery’ of the network that have few connections with other countries within the network.

Using this logic, the results of plotting Canada's NTD k-core measures in a k-core bar graph are presented in Figure 4. Figure 4 illustrates that the pattern of Canada's NTD research network k-core bar graph is not uniform across all of the bars, with increases and decreases as *k* increases. The percentage decrease of nodes left in the core is higher than approximately 80% of the other increments; which indicates dense connections among the subset of the twelve countries in the 7k-core countries and indicates a core-periphery structure.

### The “Core” of Canada's NTD Research Network

The countries that exist within the 7k-core network appear clustered between three main world regions: North America, Northern Europe, and Africa. Of the twelve countries within Canada's 7k-core, there appears to be variation in the socio-economic status of the countries; there was a 7∶5 split in OECD and non-OECD country status core members (Table 5). All five non-OECD countries within the 7k-core are African nations, indicating a predominant grouping and connection of African nations working within Canada's core-research group. Uganda and Kenya are the African nations with the most country connections (with other OECD and non-OECD countries) per publication, with both of their NTD research activities beginning in the mid-1990s and peaking between the years 2000 and 2005. Of the sixteen publications with Ugandan authors, a majority are focused on river blindness (n = 10). The others focused on African sleeping sickness (n = 4), hookworm (n = 1), and blinding trachoma (n = 1) and are classified within the fields of infectious disease (n = 7), biochemistry (n = 4), pediatrics (n = 3), and veterinary sciences. Nine of the publications with Ugandan researchers included researchers from Canada and one other country, and seven of the publications included researchers from at least four to six different countries (a majority including co-authors from the United States, Switzerland, and Germany and the others including a mix of neighboring African nations including Ghana, Kenya, Tanzania and others including Cameroon, Côte d'Ivoire, and Congo). The nine publications with Kenyan authors specialized in African sleeping sickness (n = 6). The other NTD areas of focus included Leishmania (n = 2) and riverworm (n = 1) in the fields of medicine (n = 2), pediatrics and child health (n = 2), biochemistry (n = 1), infectious disease (n = 1), immunology (n = 1), endocrinology (n = 1), and veterinary sciences (n = 1). A majority of the Kenyan publications (n = 6) included researchers from Canada and one other country, and three of them included Canada and three other countries (n = 1) or five other countries (n = 2). Relative to publications with Ugandan researchers, publications with Kenyan researchers had a greater co-authorship grouping with OECD nations including the United States, the United Kingdom, Switzerland, and Germany. Uganda and Nigeria were the only other African nations grouped with Kenya.

**Table 5:** Canada's NTD co-authorship network by countries in the 7k-core (n = 12).

Country	First Auth Count	First Auth Rank	All Auth Count	All Auth Rank	All NTD Pub	All NTD Pub Rank	SJR NTD Avg	SJR Rank
Canada	879	1	2888	1	1079	1	0.815	1
United States	50	2	355	2	141	2	0.108	2
United Kingdom	21	3	114	3	59	3	0.04	3
France	16	4	99	4	40	4	0.031	4
Germany	11	7	54	7	23	5	0.014	6.5
Switzerland	5	10	28	9.5	16	7.5	0.01	11.5
Uganda	0	49.5	28	9.5	16	7.5	0.009	13.5
Kenya	4	13.5	23	15	9	16	0.006	17
Belgium	1	29.5	16	19.5	10	11.5	0.006	17
Cameroon	0	49.5	9	27.5	6	24	0.004	23.5
Nigeria	3	17	12	23.5	5	26	0.003	29
Ghana	0	49.5	8	29	5	26	0.003	29

The data suggest that within Canada's entire NTD research network, the OECD countries within the 7k-core network are also the most active lead and contributing authors. Of the twelve countries that constitute the 7k-core, seven fall within the 80^th^ percentile of Canada's NTD research first-author count (Canada, United States, United Kingdom, France, Germany, Switzerland, and Kenya), two fell within the 50^th^ and 70^th^ first-author percentile (Belgium and Nigeria) and the remaining three fell within the 10^th^ to 49^th^ percentile (Uganda, Cameroon, and Ghana).

A majority (85%) of the countries in the 7k-core are ranked within the top 25 countries with the greatest number of all contributing authors to Canada's NTD research. Seven of the 7k-core countries fell within the 80^th^ percentile, with the remaining countries placing over the 50^th^ percentile. Of the five African nations within the 7k-core, a total of three (Ghana, Nigeria, and Kenya) had the lowest count of contributing authors. Canada, the United Kingdom, the United States, and France had the largest number of contributing authors in the 7k-core. These results suggest that contrary to the position and rank of 7k-core OECD nations, for most 7k-core African countries, just because they are well connected does not mean they demonstrate the most authorship activity. If a conventional bibliometric analysis based on authorship activity/country were the only technique used to understand co-authorship patterns, the analysis would have neglected countries that demonstrate multiple linkages and indication of greater group engagement relative to other countries. Ten of the twelve countries (83%) within the 7k-core ranked in the top 25 countries contributing to all of Canada's NTD publications (n = 1079). Four fell into the 90^th^ percentile (United States, United Kingdom, France, and Germany); two of the countries fell within the 80^th^ percentile (Switzerland and Uganda) and the remaining fell within the 60^th^–70^th^ percentile (Cameroon, Nigeria, and Ghana). The 7k-core countries were distributed across the ranking of the country SJR NTD publication average. Five of the countries fell within the 80–90^th^ percentile rank for country NTD paper SJR average percentile (United States, United Kingdom, France, Germany, and Switzerland), four fell within the 60–70^th^ percentile (Uganda, Kenya, Belgium, and Cameroon) and two countries fell within the 50^th^ percentile (Nigeria and Ghana). These data point to a potential connection between a country's connectivity (k-core), publication production, and quality rates, a correlation that is later examined.

### The Periphery of Canada's NTD Research Network

The countries that have < = 1 tie (and are dropped by UCINET in the k-core analysis) represent countries that exist on the periphery of Canada's NTD co-authorship network. These countries (n = 16 of 62), which have only one co-authorship tie to Canada's NTD publications (the one tie being to Canada), are outlined in Table 6 alongside the 2K-Core network. The 2K-core represents countries that have a maximum relationship with two countries (Canada plus one other country). In Canada's NTD co-authorship country network, four countries fit within the 2K-core, including Costa Rica, Gambia, Cuba, and Syrian Republic. Combined, the 2K-Core and the countries with less than one tie represent countries from a range of regions including Latin America and the Caribbean (n = 7), Asia (n = 5), Africa (n = 4), and Eastern Europe (n = 3) and represent countries within Canada's network within the lowest GDP percentile.

**Table 6:** Canada's NTD co-authorship network by countries in the 2k-core and 1k-core.

Country	First Auth Count	First Auth Rank	All Auth Count	All Auth Rank	All NTD Pub	All NTD Pub Rank	SJR NTD Avg	SJR Rank
2k-core (n = 4)								
Costa Rica	0	49.5	3	42.5	1	54	0.001	47.5
Gambia	1	29.5	2	50	2	41	0.001	47.5
Cuba	0	49.5	1	58.5	1	54	0.001	47.5
Syrian Arab Republic	0	49.5	1	58.5	1	54	0	58.5
1k-core (n = 16)								
Burkina Faso	0	49.5	1	58.5	1	54	0.004	23.5
Italy	0	49.5	5	36	3	33.5	0.003	29
Austria	2	20.5	12	23.5	3	33.5	0.002	36.5
Greece	1	29.5	5	36	2	41	0.002	36.5
Pakistan	1	29.5	4	38.5	1	54	0.002	36.5
Panama	0	49.5	3	42.5	2	41	0.002	36.5
Mexico	1	29.5	9	27.5	2	41	0.001	47.5
Nepal	0	49.5	2	50	1	54	0.001	47.5
Chile	0	49.5	2	50	1	54	0.001	47.5
Cambodia	0	49.5	1	58.5	1	54	0.001	47.5
Egypt	0	49.5	1	58.5	1	54	0.001	47.5
Finland	1	29.5	4	38.5	1	54	0	58.5
Malaysia	1	29.5	3	42.5	1	54	0	58.5
Zambia	0	49.5	2	50	1	54	0	58.5
Uruguay	0	49.5	1	58.5	1	54	0	58.5
Haiti	0	49.5	1	58.5	1	54	0	58.5

Four countries in the 2K-core sit within the 75% lowest percentile of first author rank. Gambia is the anomaly, ranked in the middle of the first author pack but not heavily connected with Canada's overall network.

### Is There a Correlation between K-core, Publication Count and Quality?

The network measures generated in this study help identify and characterize the groups of countries connected to Canada's NTD network and provide important insights into the composition and structure of existing relationships. The findings prompt further question as to whether collaborative research amongst countries is associated with increased research productivity and quality. To test this hypothesis, the statistical analysis software Stata10 was used to run a Pearson's correlation test on the 62 country observations (including Canada). The test was completed to identify the strength of relationship between each country's k-core rank, SJR average, total count of NTD publications, and total first author count (File S2). The Pearson's correlation test found an insignificant relationship amongst all of the variables. When the relationships were displayed in a scatter-graph it became evident that the design of the data collection resulted in the existence of an outlier, Canada. At minimum, all data had to include one Canadian author. The Pearson correlation is highly sensitive to outliers and therefore not an appropriate summary measure of the degree of relationship for this study. Rather than removing Canada from the test, a Spearman Rank Order Correlation coefficient (which is less sensitive to outliers) was run and found a strong positive correlation between the k-core and publication that was statistically significant (r = 0.80, p<0.01). A Bonferroni adjustment was then performed on the Spearman Rank Order Correlation to guard against cumulative type 1 error, and overall a positive relationship was also found among the variables (Spearman's r = 0.6937, p<0.0083). The Bonferroni adjustment found a very strong relationship between a country's k-core and total NTD publication count (Spearman's r = 0.8028, p<0.0083), a moderately strong relationship between k-core and SJR average (Spearman's r = 0.6937, p<0.008) and a positive but weak relationship between k-core and first authorship status (Spearman's r = 0.444, p<0.0083). An analysis of the implications of these tests and results is included in the subsequent discussion section.

## Discussion

The results of this study found that NTD researchers affiliated with Canadian institutions specialize (publish most) in Leishmania, African sleeping sickness, leprosy, and Chagas disease publications. Explanations for why the concentration of research activity is on these diseases are currently speculative. When research inputs are examined, the high publication volume concentrated on Leishmania and Chagas disease appears to be aligned with the higher levels of CIHR funding (relative to the other NTDs), that these diseases appear to have been receiving over the past decade. The Gabriel et al. (2010) study revealed that between 1999–2009, the greatest amount of Canada's NTD research funding from the Canadian Institute of Health Information (CIHR) was directed towards Leishmania ($28,934,502), followed by trachoma ($3,511,227), leprosy ($1,952,349), and Chagas disease ($1,685,721) [12]. These figures are based on Canadian dollars. In contrast, African sleeping sickness, the NTD that experienced the second most publishing activity, appears to have received minimal CIHR funding. Of the CIHR's $6.36 billion budget, during the ten year time period examined, a total of $29.6 million (0.4%) was allocated to research related to neglected tropical diseases. This is compared to 3.9% for HIV, 0.2% for malaria, and 0.5% for tuberculosis. While these data are important contributions to understanding Canada's NTD research platform, CIHR is one of multiple funders of Canada's global health research mandate (Examples of other Canadian global health funders include Canada's International Development Research Council, Global Health Research Initiative (GHRI), Grand Challenges, Development Innovation Fund (DIF), Canadian Public Health Research Agency and Health Canada, Canadian Collaborative for Global Health Research, Canadian Society for International Health, and the Neglected Global Disease Initiative).

The 2010 Global Funding of Innovation for Neglected Diseases Report (G-Finder), which captures data on national financial contributions to neglected disease research (including HIV/AIDS, tuberculosis, and malaria) ranked Canada fourteenth in neglected disease funding investment, compared to eighth in 2007 [28]. Canada's low 2010 score was attributed to a lack of data provided by Canadian agencies. A comprehensive financial figure of the resources Canada has provided in support of NTD research has itself been an area of neglect and underscores the need to develop and apply methods for identifying and measuring various aspects of research contributions to the field. Further research that provides the full investment picture is needed to be able to meaningful analyze Canada's financial commitment and how it correlates to research outputs and outcomes. Future analysis, for instance, could focus on a comparative analysis of the emergence of specific types of NTD research amongst institutional networks, such as Leishmania and African sleeping sickness. Examining similarities and differences between the structural characteristics of each network (such as the growth, size, density, cohesion, clustering, and positioning of institutions) may help identify areas of existing research capacity and gaps across the system that may be influencing research priorities and practices; and help inform strategic global health research innovation policy and planning.

This study found that over the past sixty years (1950–2010), a core-periphery NTD network structure at the country level has been forming in Canada. The ‘core’ network can be characterized as a dense and diverse group of countries representing a split between OECD and non-OCED countries. Although the countries on the periphery of Canada's NTD research network represent diverse geographic regions, they predominantly are non-OECD countries (including Latin American countries such as Costa Rica, Panama, Uruguay, Mexico, and Chile and South East Asian countries such as Malaysia, Nepal, Burkina Faso, and Cambodia) and fall within the lowest GDP percentile (relative to all 62 countries within the network). If the pledge by Canada and the international global health communities to build low-and-middle (LMIC) income research capacity and support research collaborations that are relevant and driven by LMIC needs is a sustainable commitment [29]; the measures and maps generated through this type of procedure, when re-run in ten years' time, should illustrate a picture with more inclusion and connections between Canada and LMIC researchers. The international attention to global health research collaborations and networks has emerged at an opportune time. The global fiscal constraints and changing economic circumstances are prompting trends whereby North-American and European countries are merging and forming alliances with companies in emerging markets (such as Brazil, India, and China). The IMS Health, for example, estimates that between 2005 and 2015, the pharmaceutical product market share of the 17 highest growing emerging markets will increase from 12%–28% [30].In the next decade, if targeted correctly, this new orientation may have trickle effects to public institutions and neighboring LMICs and open opportunities for NTD research, development and innovation (or at the very least, other global health fields that are relevant to local market needs).

These findings substantiate limitations of solely counting on the use of bibliometrics such as ‘publication activity’ or ‘co-authorship rate’ as indicators of research performance. In this study, generating network measures and running a k-core network analysis indicated that despite the high publishing activity of non-OECD countries (including Brazil, India, Peru, Iran, and Vietnam) they are not a part of Canada's NTD research 7k-core network; well-connected countries. African nations, such as Uganda, Kenya, Ghana, Cameroon, and Nigeria appear to be the predominating non-OECD countries in the k-core. In particular, Uganda is a non-OECD outlier, demonstrating the highest number of country collaborations with both OECD nations and other neighboring African nations in the field of African sleeping sickness and Leishmania. These collaborations have largely occurred in the past ten years (2000–2005). For interested funding agents, further investigating the political and economic conditions that have prompted Uganda's research collaboration activity with Canada and other non-OECD countries and the outcomes of the work conducted to date would be a step in the right direction for targeting and harnessing Canada's existing global health research partnerships.

Existing literature suggests that researchers involved in more diverse collaborative networks are more productive in terms of producing publications and seeking research grants [31]–[33]. While this study was not designed to test that hypothesis directly (in terms of research grant activity), it did find that a majority of the OECD countries that fell within Canada's 7k-core (most connected countries) ranked in the top percentile of lead authorship, author count, publication count, and country SJR average. Kenya was one of the few African countries in the 7k-core that also demonstrated such results. Kenya fell within the 80^th^ percentile of first authors and 70^th^ percentile of publication contribution against all other countries and was the only African country within the 70^th^ percentile for the country SJR average. Implications of the differences found in this study between the publication ‘productivity’ of OECD and non-OECD nations are well documented within the literature [34].

Relative to other Canadian research fields, Canada's NTD research country collaboration rate appears comparable. The Association of Universities and Colleges of Canada (AUCC) reports that Canadian researchers have increased rates of co-publishing with authors from emerging and developing countries from 3.4% in 1992 to 6.4% in 2003 [35]. More than 40% of academic publications by Canadian authors have co-authors from other countries. This is twice the rate from fifteen years ago. Canadian universities are reportedly taking more initiative to “internationalize” their campuses through supporting technology transfer agreements, research networks and other cooperative arrangements. If international collaboration is a strategic global health research priority for Canada – then this data could set a baseline for our international research collaboration rate in the specific field of NTDs.

In the field of tropical medicines, the idea that research agendas in the developing world have historically been dictated by the richer countries has become a truism. Many researchers argue that collaborations between researchers from the “North” and “South” need to be transformed into research partnerships that ensure mutual learning and institutional capacity building [36], [37]. The call for partnerships is supported by arguments that suggest that resource-limited countries have historically been faced with a lack of research funding, poor research facilities, and limited career opportunities, which have often resulted in brain drain. Mutually beneficial and equitable partnerships are considered a required mechanism for stimulating a conducive and sound environment for research in developing countries. The hope is that research partnerships and networks may be a critical lever for increasing the productivity and quality of research and evidence that is relevant to local needs and ultimately results in positive health outcomes for these countries and their neighboring regions. Ynalvez and Shrum [34] looked at the association between scientific collaboration and publication productivity in resource-constrained research institutes in a developing country and found that publication productivity and quality are significantly linked to professional network factors. In the case of Canada's NTD research network, there is an indication that the more a country is connected to other countries, the greater the publication activity and higher quality of research that is produced. Further testing of predictors such as OECD status or GDP rank and modeling on other datasets are welcomed and needed to substantiate these claims.

**Figure 5:**
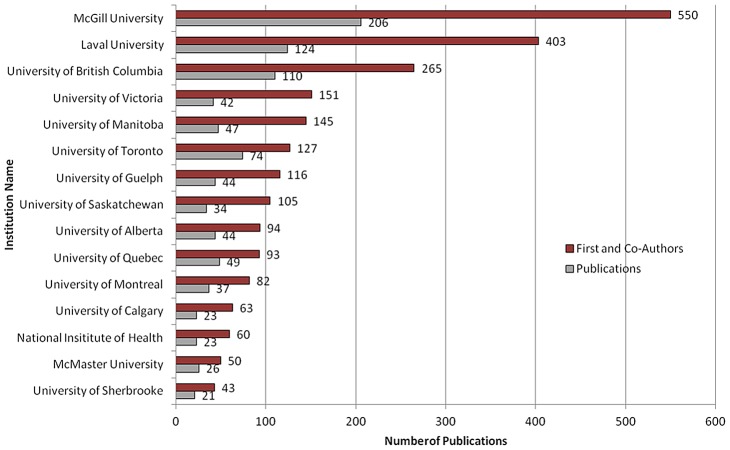
Canada's NTD research. Top 15 institutions by authorship and publication activity.

Limitations in the use of bibliometrics to generate tropical medicine publication data have been addressed in the literature, such as an apparent selection bias and exclusion of Latin American and African journals within indexes of dominating databases (Medline, Embase, and ISI Web of Knowledge) that privileged North American and European authors [38], [39]. To address these limitations, databases like SciELO, a result of a World Health Organization and Pan American Health Organization (PAHO) collaboration, have been created. SciELO now includes 17 journals in the field of tropical and infectious diseases from Argentina (1), Brazil (8), Chile (4), Cuba (1), and Venezuela (2) [40]. However, the SciELO itself has limitations for the purposes of collecting bibliometric data. When this study was conducted, the search functionality of SciELO did not allow for co-author search, a key design feature of this work. Instead, the Scopus database, which includes 55% more papers on tropical medicine than ISI Web of Science and is the largest selection of journals from more countries with a greater variety of fields in tropical medicine, was selected and used [25]. A further limitation of this study design, due to resource limitations, was the exclusion of studies published in other languages; such as French. Despite this limitation, the bibliometric data showed large representation of researchers from across Canada's bilingual Quebec universities and research institutes who have published in English. These include first and co-authors from McGill University (550 authors; 206 publications), University of Laval (403 authors; 124 publications), University of Quebec (93 authors; 49 publications), and University of Montreal (82 authors, 37 publications); which represents 39% of all authorship and 38.5% of all publications (Figure 5). A future study, with dedicated resources for translation support, could re-run the procedure and expand this network analysis to include NTD publications written in all languages.

This study is also limited by the old-adage that bibliometric data are only as strong as the individuals who input and maintain them. Prior to the 1980s, Scopus, similar to many academic databases are limited in their publication coverage; which likely explains the low publication production count before the 1980s. This analysis was also dependent on the expertise and subjectivity of the experts hired to classify academic publications into common categories, a process that, although subjective, supports researchers in identifying and describing patterns and trends in research communities. For instance, Scopus invests significant resources into developing universal classifications to categorize types of research publications. Similar to any research that involves archival data, the validity of the network data is highly dependent on and sensitive to the capabilities of the databases and the individuals inputting the information; a limitation that should be considered when interpreting the results of this work.

When interpreting the results, it is also important to consider the main difference between network data and conventional data; network data focuses on actors and relations; whereas, conventional data focuses on actors and attributes. The actors in non-network studies are largely the result of independent probability sampling. In contrast, network data often include all actors who occur within some (often naturally occurring) boundary [27]. In this study, the network is naturally shaped and bounded by the NTD publication data that was generated through the bibliometric search. In this way, the size of the international collaboration dataset used to understand the core-periphery of the network is appropriate given this study represents a bounded (and limited) network of Canadian NTD publication authors (English-language) and their connection with co-authors. Future work could expand the network by surveying Canadian and international authors identified within this bounded network and ask them to identify a ‘free-list’ of who else they've collaborated with on NTD research.

### Conclusion

The objective of this analysis is to understand Canada's historic contribution to NTD publication activity and the core-periphery structure of Canada's research network. It also discusses the features and characteristics of the international co-authoring partners and types of NTD research that is being conducted. In this study, the bibliometric information, country collaboration rates, and k-core measures used to analyze the authorship patterns and core-periphery of the network provide evidence that researchers in Canada currently remain the overall gatekeepers of the NTD research Canada generates. Gatekeepers represent individuals or institutions predominantly responsible for setting agendas [41]. Of Canada's 1079 NTD publications, 64.8% (n = 700) do not include international authors. This appears in spite of commitments such as the 2008 Bamako Call whereby Canada aims to ensure that partners from the South are leading the global health research agenda [42].

The methodology and findings from this study provides new insight to multiple stakeholders interested in evidence and trends in international research networks. Researchers, public and private funders, and not-for-profit organizations and policy makers may use the methodology or study findings to conduct focused case studies that measure, map, and assess the scientific activities of leading NTD researchers, institutions, and/or funding agents. All in all, this work substantiates the call for future analysis that looks at trends in specific NTD areas and the structures, actors, and factors that are supporting or impeding Canadians from partnering and publishing with LMIC researchers to further advance improvements in NTD research and development.

## Supporting Information

File S1 **Scopus NTD search string.** This file contains the list of search strings, developed for the twelve NTDs that were used to carry out this research.
(TIF)

File S2 **Stata 10 analysis—strength of relationship between each of Canada's NTD research network country's k-core rank, SJR average, total count of NTD publications and total first author count.** This file contains the results of the statistical analysis tests used in this study.
(TIF)
