# Supplementary figures and images for: Canada's Neglected Tropical Disease Research Network: Who's in the Core—Who's on the Periphery?
Source: PLoS Negl Trop Dis. 2013 Dec 5;7(12):e2568. doi: 10.1371/journal.pntd.0002568 (PMC3854962; doi:10.1371/journal.pntd.0002568)

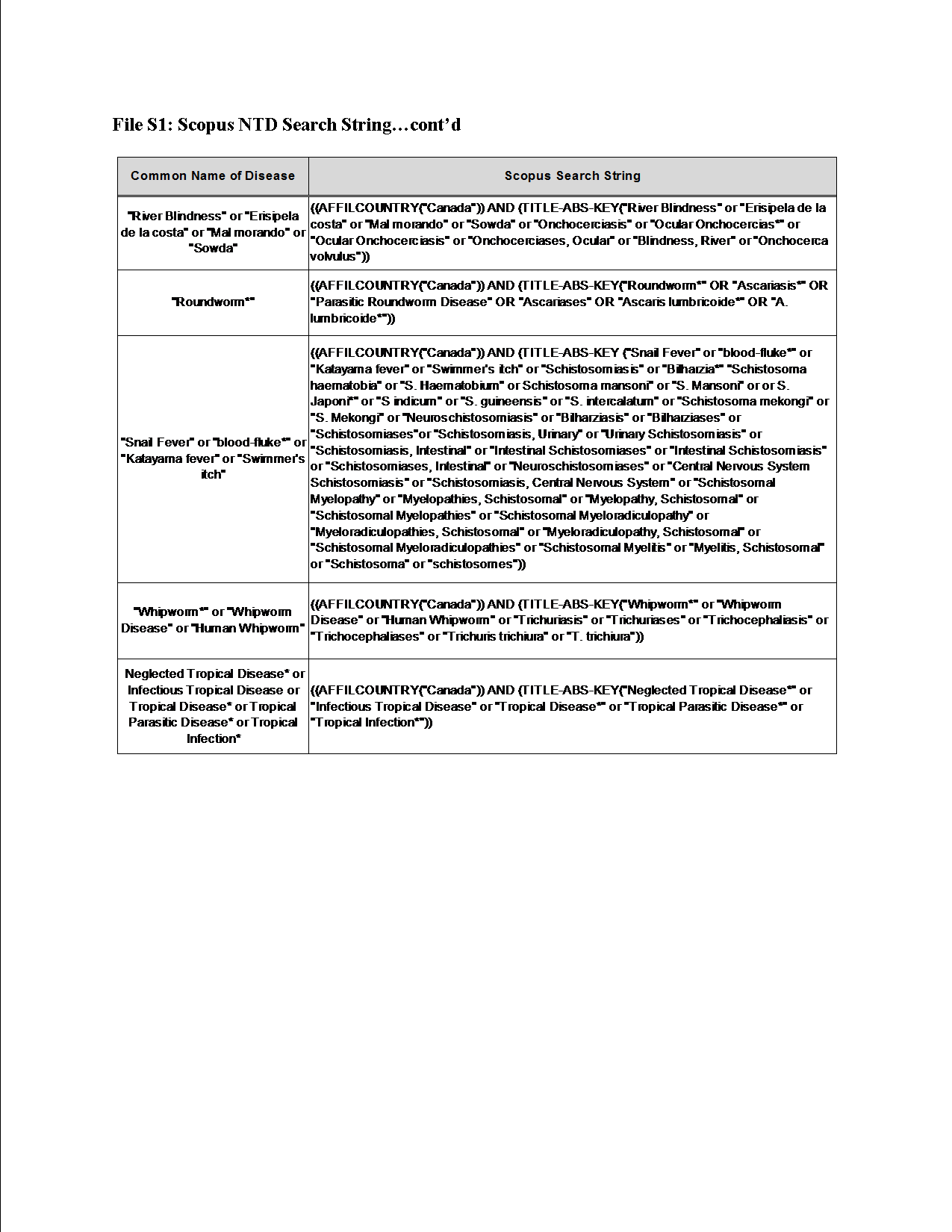

Supplement: File S1 — Scopus NTD search string. This file contains the list of search strings, developed for the twelve NTDs that were used to carry out this research. (TIF) [file pntd.0002568.s001.tif]

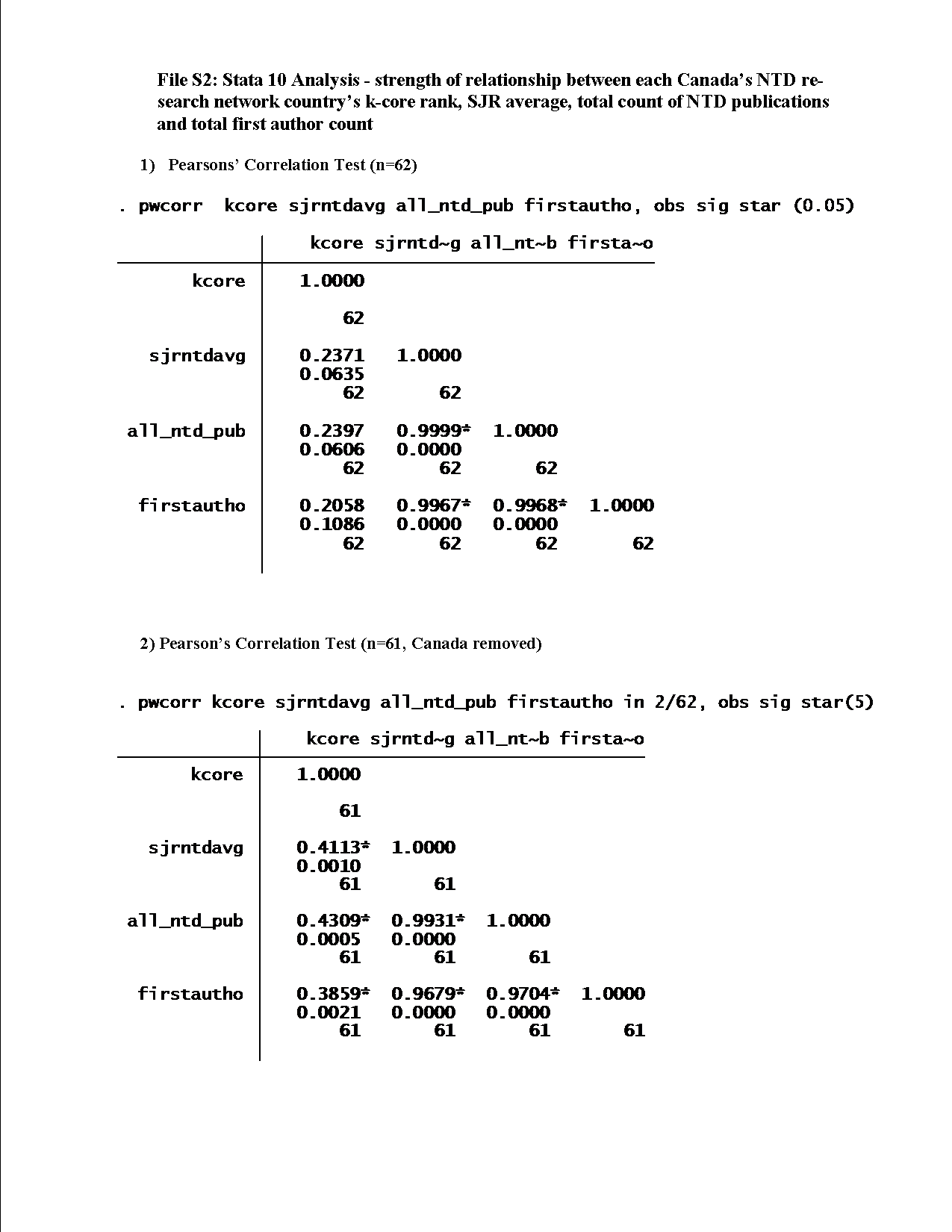

Supplement: File S2 — Stata 10 analysis—strength of relationship between each of Canada's NTD research network country's k-core rank, SJR average, total count of NTD publications and total first author count. This file contains the results of the statistical analysis tests used in this study. (TIF) [file pntd.0002568.s002.tif]
